# High Risk of Hip and Spinal Fractures after Distal Radius Fracture: A Longitudinal Follow-Up Study Using a National Sample Cohort

**DOI:** 10.3390/ijerph18147391

**Published:** 2021-07-10

**Authors:** Hyo-Geun Choi, Doo-Sup Kim, Bumseok Lee, Hyun Youk, Jung-Woo Lee

**Affiliations:** 1 Department of Otorhinolaryngology-Head & Neck Surgery, Hallym University College of Medicine, Anyang 14068, Korea; hgchoi@hallym.or.kr; 2 Hallym Data Science Laboratory, Hallym University College of Medicine, Anyang 14068, Korea; 3 Department of Orthopaedic Surgery, Wonju College of Medicine, Yonsei University, Wonju 26426, Korea; dskim1974@yonsei.ac.kr (D.-S.K.); bumseok@yonsei.ac.kr (B.L.); 4 Department of Emergency Medicine, Wonju College of Medicine, Yonsei University, Wonju 26426, Korea; yhmentor@yonsei.ac.kr; 5 Bigdata Platform Business Group, Wonju Yonsei Medical Center, Yonsei University, Wonju 26426, Korea

**Keywords:** cohort study, follow-up studies, radius fractures, hip fractures, spinal fractures

## Abstract

The purpose of the present study was to estimate the risk of hip and spinal fracture after distal radius fracture. Data from the Korean National Health Insurance Service—National Sample Cohort were collected between 2002 and 2013. A total of 8013 distal radius fracture participants who were 50 years of age or older were selected. The distal radius fracture participants were matched for age, sex, income, region of residence, and past medical history in a 1:4 ratio with control participants. In the subgroup analysis, participants were stratified according to age group (50–59, 60–69, or ≥70 years) and sex (male or female). Distal radius fracture patients had a 1.51-fold and 1.40-fold higher incidence of hip fracture and spinal fracture in the adjusted models, respectively. Among males, patients of all ages had a significantly higher incidence of hip fracture, and those who were 50 to 69 years of age had a significantly higher incidence of spinal fracture. Among females, those older than 70 years had a significantly higher incidence of hip fracture, and patients of all ages had a significantly higher incidence of spinal fracture. Previous distal radius fracture has a significant impact on the risk of subsequent hip and spinal fractures.

## 1. Introduction

Distal radius fracture is one of the most common types of fractures, and the incidence of this fracture appears to be increasing [1]. Between 2008 and 2011, the number of osteoporosis-related distal radius fractures increased by 31.6%, and total annual healthcare costs increased 45.0% among Koreans [2]. Distal radius fracture can be an important cause of mortality or loss of independence in the elderly population. Wrist fractures contribute to clinically important functional decline in older women [3]. Furthermore, distal radius fractures are associated with a significant increase in mortality [4,5,6,7,8].

Previous fractures are known to increase the risk of subsequent fractures [9,10]. However, there are inconsistencies among studies regarding the association of distal radius fracture with hip and spinal fractures. One cohort study reported that primary wrist fractures were not significantly associated with subsequent hip fractures (adjusted hazard ratio (HR) 1.29; 95% confidence interval (CI) 0.88–1.89, *p* = 0.19), and the 10-year probability of any recurrent fracture was substantially lower after a primary wrist fracture (HR 14.2; 95% CI 11.9–16.5) than after other osteoporotic fractures [11]. An observational cohort study of hip, shoulder, and wrist fragility fractures showed that the risk of a second fracture in the year following a wrist fracture was similar to the risk after a hip or shoulder fracture [12]. In contrast, wrist fractures are associated with increased subsequent hip fractures (HR 1.29–3.45, relative risk (RR) 1.4–3.26, relative hazard (RH) 1.54–2.27) [13,14,15,16,17,18,19], spinal fractures (odds ratio (OR) 1.39) [20], and both types of fractures (hip; HR 1.50, RR 1.9, OR 1.80–1.85, standardized incidence ratio (SIR) 1.4–2.7, fracture risk 1.8–6.7/1000, spine; HR 1.48, RR 1.9, OR 1.61–1.71, SIR 1.5–10.7, fracture risk 1.5–3.6/1000) [21,22,23,24,25].

Although previous studies have reported the risk of subsequent fractures, many of the previous studies were cross-sectional mailed surveys, case–control studies, or retrospective reviews of case records. The variations in cross-sectional study designs, the study populations, and confounders left unaccounted for could explain the conflicting results of prior studies. Among previous studies, five relied on a survey of patients [19,20,22,23,24], six did not include male patients [11,15,19,20,22,24], three did not include patients younger than sixty-five years old [12,19,20], eight did not divide the groups by sex and age [11,12,14,18,19,20,21,23], and four did not include enough data to estimate the HR or RR with a 95% CI [12,21,22,25]. Additionally, most articles were based on information from participants collected before 2000 [11,15,16,17,18,19,20,21,22,25].

We performed a large-scale longitudinal follow-up study using national cohort data and performed subgroup analyses according to age and sex. Additionally, we used relatively up-to-date data from 2002 and reported the adjusted HRs with the 95% CI. To the best of our knowledge, ours is the first study to demonstrate the risk of subsequent hip and spinal fractures after distal radius fracture using a national cohort, with subgroups stratified by age and sex. The purpose of the present study was to estimate the risk of hip and spinal fractures after distal radius fracture using the National Sample Cohort through a longitudinal follow-up study design. Distal radius fracture participants who were 50 years of age or older were matched for age, sex, income, and region of residence in a 1:4 ratio with control participants, and they were followed up for a minimum of 3 years and a maximum of 12 years.

## 2. Materials and Methods

### 2.1. Study Population and Data Collection

The ethics committee of Hallym University (2014-I148) approved the use of these data. The need to obtain written informed consent was exempted by the Institutional Review Board. Data from the Korean National Health Insurance Service—National Sample Cohort (NHIS-NSC) were collected and classified in the same manner as in a previous study [26,27]. The Korean National Health Insurance Service has information on the entire Korean population (~50,000,000). Among the records, 2% of participants (approximately 1,000,000) were selected directly to reflect the Korean population. The direct sample was stratified for age, sex, income, and region of residence. Access to the NHIS can be obtained through the National Health Insurance Sharing Service home page (http://nhiss.nhis.or.kr, accessed on: 1 March 2017), which provides a document on the method of extraction and validation of the sampling method; however, no English version is available. The representativeness and validity of this sample database was confirmed by comparing estimates based on the sample data and the entire population [28].

### 2.2. Study Design

We diagnosed distal radius fracture using the ICD-10 code over a specific duration and selected participants who were 50 years of age or older. Then, the distal radius fracture participants were matched in a 1:4 ratio with the control group. Participants with previous hip or spinal fracture were excluded, and those for whom sufficient matching controls could not be identified were also excluded. Then, we found hip or spinal fractures through longitudinal follow-up.

### 2.3. Participant Selection

Out of 1,125,691 cases with 114,369,638 medical claim codes, we included distal radius fracture patients from March 2002 through December 2011. Among them, we selected the participants who were 50 years of age or older when the distal radius fracture occurred (n = 8013).

The distal radius fracture participants were matched in a 1:4 ratio with participants (control group) who were never diagnosed with a distal radius fracture from 2002 through 2013 among the original cohort. Participants who were diagnosed with a hip fracture or spinal fracture before the distal radius fracture occurred were excluded (n = 264). The cases and controls were matched for age group, sex, income group, region of residence, and past medical histories (hypertension, diabetes mellitus, and dyslipidemia). To prevent selection bias when selecting the matched participants, the control group participants were sorted using a random number order, and they were then selected from top to bottom. It was assumed that the matched control participants were involved at the same time of each matched distal radius fracture participant (index date). Therefore, the members of the control group who died before the involvement of the matched distal radius fracture participants were excluded. In the control group, the participants who were diagnosed with a hip fracture or spinal fracture before the index date were excluded. The distal radius fracture participants for whom we could not identify enough matching participants were excluded (n = 69).

The distal radius facture patients and control participants were followed up to death or 31 Dec 2013. The mean follow-up time was 78.3 month (standard deviation (SD) = 32.2) for distal radius fracture patients and 78.4 months (SD = 32.1) for control participants.

### 2.4. Variables

#### 2.4.1. Independent Variable

Distal radius fractures were diagnosed using an ICD-10 code (fracture of lower end of radius (S525)).

#### 2.4.2. Covariate Analysis

Age groups were divided into 5-year intervals: 50–54, 55–59, 60–64…, and 85+ years old. Income and region of residence were determined following our previous studies [29,30].

The past medical histories of the participants were evaluated using ICD-10 codes, following our previous studies [26,31].

#### 2.4.3. Dependent Variable

In this study, hip fracture was defined as the fracture of the head and neck of the femur (S720), pertrochanteric fracture of the femur (S721), or subtrochanteric fracture of the femur (S722). Spinal fractures were defined as fractures of the thoracic vertebrae (S220) or fractures of the lumbar vertebrae (S320). Cervical spinal fracture was not included because according to our previous studies, this fracture is not affected by osteoporotic fractures [27,32]

### 2.5. Statistical Analyses

To analyze the HRs for hip and spinal fractures in patients with distal radius fractures, a stratified Cox proportional hazard model was used. In this analysis, crude (simple) and adjusted (ischemic heart disease, cerebral stroke, depression, and osteoporosis histories) models were used. The 95% CI was calculated. The analysis was stratified for variables such as age, sex, income, region of residence, hypertension, diabetes mellitus, and dyslipidemia. In the subgroup analysis, participants were stratified according to age group (50–59 years, 60–69 years, or ≥70 years) and sex (male or female). From the bivariate analysis, distal radius fracture was the independent variable, and hip fracture and spine fracture were dependent variables. Other variables such as age, sex, income, region of residence, hypertension, diabetes mellitus, and dyslipidemia were covariate.

To analyze the cumulative probability of hip and spinal fractures, a Kaplan–Meier analysis was used.

Two-tailed analyses were conducted, and *p* values less than 0.05 indicated significance. The results were statistically analyzed using SPSS v.22.0 (IBM, Armonk, NY, USA).

## 3. Results

**Figure 1:**
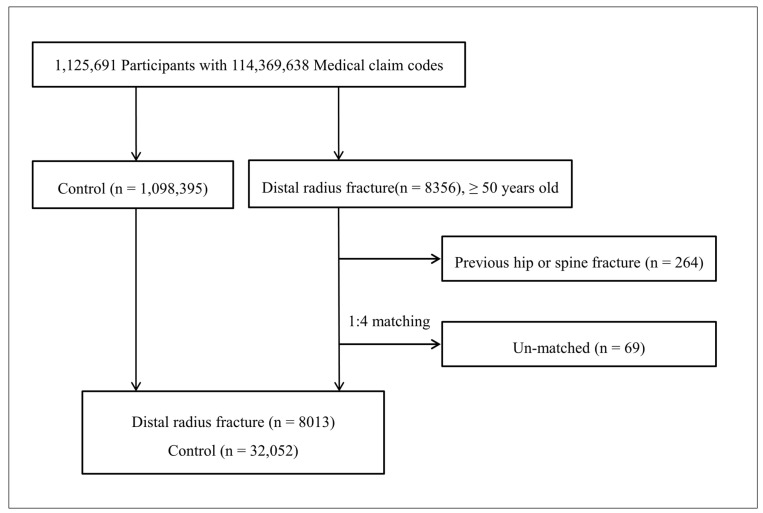
A schematic illustration of the participant selection process that was used in the present study; 8013 distal radius fracture patients and 32,052 control participants were included.

Consequently, 1:4 matching resulted in the inclusion of 8013 distal radius fracture patients and 32,052 control participants (Figure 1). These distal radius fracture patients and control participants were followed up for a minimum of 3 years and a maximum of 12 years. During follow-up, we found 923 cases of hip fractures and 2979 cases of spinal fractures. This is a follow-up study using a national sample cohort.

**Figure 2:**
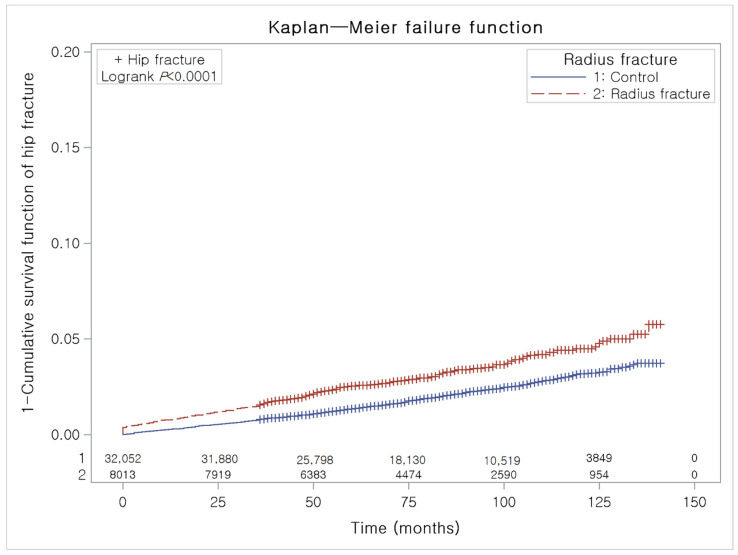
A Kaplan–Meier analysis of the incidences of hip fracture in the distal radius fracture group and the control group. During the follow-up period, the cumulative incidence of hip fracture in the distal radius fracture group was significantly higher than that in the control group.

**Figure 3:**
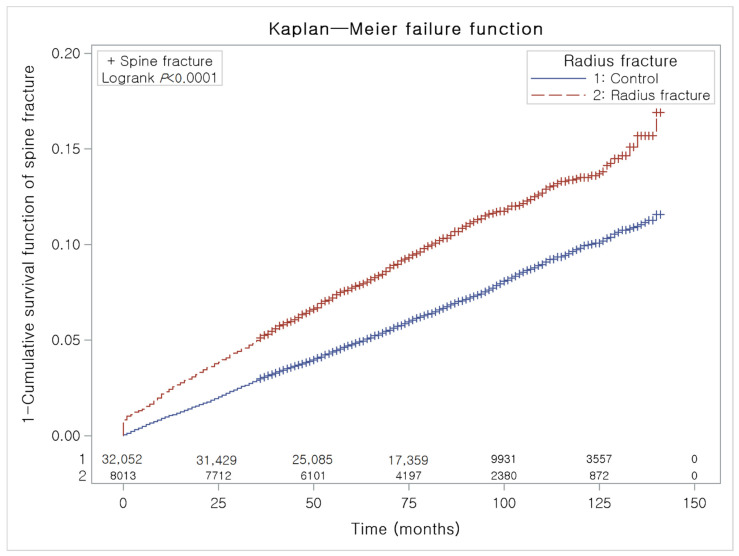
A Kaplan–Meier analysis of the incidences of spinal fracture in the distal radius fracture group and the control group. During the follow-up period, the cumulative incidence of spinal fracture in the distal radius fracture group was significantly higher than that in the control group.

Age, sex, level of income, and region of residence were matched between the distal radius fracture patients and the control participants (Table 1). The past medical histories (hypertension, diabetes mellitus, and dyslipidemia) were also matched. The mean follow-up was 78.3 months (standard deviation (SD) = 32.2) in the distal radius fracture group and 78.4 months (SD = 32.1) in the control group. During the follow-up period, a difference in the occurrence of hip fractures between the distal radius fracture group and the control group was observed (Figure 2). Additionally, a difference in the occurrence of spinal fractures between the distal radius fracture group and the control group was observed (Figure 3). The crude and adjusted HRs for hip fracture were 1.61 (95% CI = 1.40–1.86, *p* < 0.001) and 1.51 (95% CI = 1.30–1.74, *p* < 0.001), respectively (Table 2). The crude and adjusted HRs for spinal fracture were 1.55 (95% CI = 1.43–1.6, *p* < 0.001) and 1.40 (95% CI = 1.29–1.52, *p* < 0.001), respectively.

When categorizing patients according to age (50–59 years, 69–69 years, ≥70 years) and sex, the results were slightly different depending on sex. Among males, patients of all ages had significantly higher incidences of hip fracture (adjusted HR = 5.81, 3.23, 3.42, respectively), and those 50–59 and 60–69 years of age had significantly higher incidences of spinal fracture (adjusted HR 3.00, 2.37, respectively) (Table 3). Among females, those over 70 years of age had a significantly higher incidence of hip fracture (adjusted HR = 1.45), and patients of all ages had significantly higher incidences of spinal fracture (adjusted HR = 1.78, 1.33, 1.27, respectively) (Table 3). Moreover, the adjusted HRs trended to be slightly higher in men than in women regardless of age; however, the 95% CIs mostly overlapped.

## 4. Discussion

The purpose of the present study was to estimate the risk of hip and spinal fracture after distal radius fracture using the National Sample Cohort. Distal radius fracture participants who were 50 years of age or older were matched for age, sex, income, and region of residence in a 1:4 ratio with control participants, and they were followed up for a minimum of 3 years and a maximum of 12 years. Previous distal radius fracture has a significant impact on the risk of subsequent hip and spinal fractures.

We report the risk of hip and spinal fractures after wrist fracture using a large, national cohort of older adults. The incidences of secondary fractures were increased after wrist fractures. The risk of hip and spinal fractures differed according to sex and age group. The incidence of hip fractures increased in males of all ages but only in women over 70 years old. Conversely, the incidence of spinal fractures increased in men younger than 69 years old but in women of all ages.

Age and sex have a pronounced effect on the incidence rates of distal radius fractures in the elderly population [1]. The incidence of distal forearm fractures is noticeably lower in men than in women, with a male-to-female ratio of 1:4 [6]. The mean age-specific incidence increased in all age groups, and the incidence of distal radius fractures steadily increased in men and women. Despite differences in the incidence of distal radius fractures in men and women, the number of articles comparing the sexes in terms of the occurrence of secondary fractures is small. Our paper stratifies the HRs not only by sex but also by age group.

The mechanism by which previous distal radius fractures increase the risk of subsequent hip and spinal fractures is unknown. Common fractures may be a marker for some unknown factors, such as deficient bone strength or quality [33]. In patients with distal radius fracture, impaired skeletal strength is likely to be present [13,34]. However, the increased future fracture risk after wrist fracture (RR 2.4) is largely independent of peripheral bone mineral density (BMD), according to a survey study (RR 2.1 after adjusting BMD) [24]. Prior fractures might also indicate the presence of nonskeletal factors that increase the risk of fracture, such as an increased frequency of falls or reduced protective responses [35]. Patients with wrist fractures might have weakened hand strength and worse postural sway, which result in subsequent hip fractures during falls [14].

Our finding (an adjusted HR of 1.62 for subsequent hip fractures) was consistent with those of previous studies, most of which found similar incidence rates, with HRs ranging from 1.29 to 3.45 [11,14,23] and RRs ranging from 1.4 to 3.26 [13,15,16,17,18,19]. However, most of these studies were performed in Western countries, and data from Asian patients have rarely been reported. A recent study in Taiwan found that the HR of hip fracture in relation to a previous distal radius fracture was 3.45 (95% CI = 2.59–4.61) [14], however, we cannot compare their results to ours given the different study designs; their study had a 1-year follow-up period and did not categorize patients according to age. The adjusted HR for subsequent spinal fracture in our study (1.54) was similar to those in previous studies, with reported HRs (1.48) [23], RRs (1.3) [24], and ORs (1.39–1.71) falling in the same range [20,22].

In most previous papers, the risk of subsequent hip fracture has been shown to increase with age. Chen et al. reported that patients aged >60 years had the highest HR (8.67, 95% CI = 4.51–16.7) [14], and Gunnes et al. reported that patients aged >70 years had a higher OR (1.95, 95% CI = 1.49–2.54) [22]; however, neither study distinguished groups by sex. In two studies that reported both men and women, the fracture rate [12] and lifetable estimates [25] increased with age (66–74, 75–84, >85 years) in both sexes. In our study, the adjusted HRs for subsequent hip fracture were significantly higher in all male patients and in female patients over 70 years of age. Our findings are in line with previous findings that hip fractures increase with age in women. Among women sustaining a hip fracture, the adjusted RR did not differ significantly by age group (stratified by <65 vs. ≥65 years) [24] in one study but, in others, women 70–79 years of age had an increased RR of 2.2 (95% CI = 1.2–3.7) [16], those 60–79 years of age had an increased RR of 1.9 (95% CI = 1.3–2.6) [15], and the fracture rates increased with age (<60, 60–69, 70–79, >80 years) [23].

Unlike in the present study, a previous study showed that the SIRs were higher among men <70 years and among women >70 years [21], and the RH in women was independent of age while that in men was more pronounced in the younger age groups (40–49 years; RH = 5.04 [95% CI = 0.56–45.13]) [17]. Although in our study the HR was higher in all age groups among men, the overall HRs were higher in women. In a meta-analysis, the RR of hip fracture after a wrist fracture was 1.53 (95% CI = 1.34–1.74) in women and 3.26 (95% CI = 2.08–5.11) in men who were at least 50 years old [13]. These findings support the concept that wrist fractures are an early and sensitive marker of male skeletal fragility [34]. As few studies have been reported on male patients, previous results are difficult to compare with our study. More research will be needed for male patients.

The incidence of spinal fractures according to age varies among studies. In a paper that reported both men and women, the lifetime estimates of spinal fracture increased with age (66–74, 75–84, >85 years) in both sexes [25]. In studies with women who sustained spinal fractures, the adjusted RR did not differ significantly by age group (stratified by <65 vs. ≥65 years) [24] in one study, but women aged ≤70 years had a higher OR in another study (1.67, 95% CI = 1.19–2.34) [22], and fracture rates were increased with age (<60, 60–69, 70–79, >80 years) [23] in another study. In this study, for subsequent spinal fractures, male patients who were 50–69 years of age had a significantly higher incidence, and all female patients had a significantly higher incidence. These findings are in accordance with the reports by Cuddihy; the standardized incidence of spinal fracture was higher among men <70 years and women >70 years [21]. In summary, the RR of spinal fracture after wrist fracture was 1.7 (95% CI = 1.4–2.1) for peri/postmenopausal women and 7.2 (95% CI = 3.6–14.6) for other populations [35]. More research will be needed based on age group with HRs or RRs with the 95% CI.

As we used data from the Korean Health Insurance Review and Assessment Service (HIRA), which includes data from all citizens of Korea, we did not miss any participants due to loss to follow-up. Therefore, the strength of this study lies in the fact that the results accurately represent the entire Korean population, including all sexes. While many previous studies focused on elderly adult patients or women of advanced age, the present study enrolled patients who were 50 years or older. The control participants were randomly selected and matched for age, sex, income, and region of residence to avoid possible confounding effects [36]. As income and region of residence determine the availability of medical care, the matching of these variables was important, and income was accurately collected based on the Korean NHIS data. Furthermore, an adjusted hazard model was used to minimize confounding by age, sex, income, region of residence, and the histories of hypertension, diabetes, dyslipidemia, ischemic heart disease, and stroke. Finally, all wrist fracture patients were defined as presenting with a fracture of the lower end of the radius (ICD-10 codes: S525).

There were several limitations of this study. First, we used health insurance claim data; hence, there could be some inaccuracies within the data and results. Some such inaccuracies are inevitable with large cohort databases such as the NHIS; however, due to the large dataset, the results can still be meaningful. Second, we followed up the study for only three years, and we cannot know the history of fracture before the study period. However, in most fracture cases, it is thought that the patient visited the hospital because access to medical care in Korea is effortless. Third, some potential confounders for fracture risk, including smoking, obesity, and body mass index, could not be considered in this study due to the lack of information in the NHIS-NSC database. Additionally, BMD of distal radius fracture patients was not available in the NHIS-NSC data. Blood levels of estradiol and vitamin D3 may also affect hip fracture but could not be tested [37,38]. Fourth, there is no information about the timing of subsequent fractures. Fifth, as traffic accidents were excluded, a large number of accidents would have been excluded. However, our data could include high-velocity injuries from personal mobility rather than traffic accidents. Sixth, the heterogeneity of distal radius fractures and treatment modalities [39] could affect the association with subsequent fractures. Seventh, there is a possibility of selection bias because we excluded a small group for which it was challenging to find a control group. However, that number was not significant; the number of exclusions in those over 85 years old was 69. Eighth, there were limitations in the statistical calculations because the stratified Cox model is not used to analyze matched data. Lastly, we excluded 264 patients with a simultaneous or earlier occurrence of spine or hip fractures, about 3% of the sample.

Nonetheless, this cohort study contributes valuable information regarding the risk of subsequent fractures and shows that previous distal radius fracture has a significant impact on the risk of subsequent hip and spinal fractures compared with the control group. Among individuals who sustained recurrent fractures, 31–45% did so within one year of the initial fracture [40].

## 5. Conclusions

Distal radius fracture patients had a 1.51- and 1.40-fold higher incidence of hip fracture and spinal fracture in the adjusted models, respectively. In particular, females aged 50 to 59 years had a significantly higher incidence of spine fracture.

Previous distal radius fracture has a significant impact on the risk of subsequent hip and spinal fractures.

## Figures and Tables

**Table 1:** General characteristics of participants.

Characteristics			Distal Radius Fracture		
			Fracture (n, %)	Control (n, %)	*p*-Value
Age (years)					1.000
		50–54	1297 (16.2)	5188 (16.2)	
		55–59	1598 (19.9)	6392 (19.9)	
		60–64	1469 (18.3)	5876 (18.3)	
		65–69	1423 (17.8)	5692 (17.8)	
		70–74	1035 (12.9)	4140 (12.9)	
		75–79	581 (7.3)	2324 (7.3)	
		80–84	385 (4.8)	1540 (4.8)	
		85+	225 (2.8)	900 (2.8)	
Sex					1.000
		Male	1533 (19.1)	6132 (19.1)	
		Female	6480 (80.9)	25,920 (80.9)	
Income					1.000
		1 (lowest)	84 (1.0)	336 (1.0)	
		2	722 (9.0)	2888 (9.0)	
		3	578 (7.2)	2312 (7.2)	
		4	557 (7.0)	2228 (7.0)	
		5	545 (6.8)	2180 (6.8)	
		6	662 (8.3)	2648 (8.3)	
		7	687 (8.6)	2748 (8.6)	
		8	788 (9.8)	3152 (9.8)	
		9	977 (12.2)	3908 (12.2)	
		10	1136 (14.2)	4544 (14.2)	
		11 (highest)	1277 (15.9)	5108 (15.9)	
Region of residence					1.000
		Urban	3592 (44.8)	14,368 (44.8)	
		Rural	4421 (55.2)	17,684 (55.2)	
Hypertension			4673 (58.3)	18,692 (58.3)	1.000
Diabetes mellitus			2225 (27.8)	8900 (27.8)	1.000
Dyslipidemia			2855 (35.6)	11,420 (35.6)	1.000
Ischemic heart disease			780 (9.7)	3152 (9.8)	0.788
Cerebral stroke			1526 (19.0)	5741 (17.9)	0.018 *
Depression			1099 (13.7)	3833 (12.0)	<0.001 *
Osteoporosis			3946 (49.2)	12,981 (40.5)	<0.001 *
Hip fracture			262 (3.3)	661 (2.1)	<0.001 *
	Age 50–59 y		22 (0.8)	42 (0.4)	0.004 *
	Age 60–69 y		52 (1.8)	163 (1.4)	0.122
	Age 70+ y		188 (8.4)	456 (5.1)	<0.001 *
Spinal fracture			812 (10.1)	2167 (6.8)	<0.001 *
	Age 50–59 y		141 (4.9)	261 (2.3)	<0.001 *
	Age 60–69 y		310 (10.7)	846 (7.3)	<0.001 *
	Age 70+ y		361 (16.2)	1060 (11.9)	<0.001 *

* Chi-square test, significance at *p* < 0.05.

**Table 2:** Crude and adjusted hazard ratios (95% confidence interval) of distal radius fracture for hip and spinal fracture in total participants (n = 40,075).

Fracture		Distal Radius Fracture			
		Crude †	*p*-Value	Adjusted †‡	*p*-Value
Hip fracture			<0.001 *		<0.001 *
	Yes	1.61 (1.40–1.86)		1.51 (1.30–1.74)	
	No	1.00		1.00	
Spinal fracture			<0.001 *		<0.001 *
	Yes	1.55 (1.43–1.6)		1.40 (1.29–1.52)	
	No	1.00		1.00	

* Cox-proportional hazard regression model, significance at *p* < 0.05; † stratified model for age, sex, income, region of residence, hypertension, diabetes mellitus, and dyslipidemia; ‡ adjusted model for ischemic heart disease, cerebral stroke, depression, and osteoporosis histories.

**Table 3:** Subgroup analysis of adjusted hazard ratios (95% confidence interval) of distal radius fracture for hip and spinal fracture according to age.

Fracture		Distal Radius Fracture					
		50–59 y (n = 3470)		60–69 y (n = 2570)		70+ y (n = 1625)	
		Adjusted †‡	*p*-value	Adjusted †‡	*p*-value	Adjusted †‡	*p*-value
Hip fracture							
Male			0.002 *		<0.001 *		<0.001 *
	Yes	5.81 (1.94–17.36)		3.23 (1.70–6.16)		3.42 (1.95–6.00)	
	No	1.00		1.00		1.00	
Female			0.198		0.521		<0.001 *
	Yes	1.51 (0.80–2.84)		0.88 (0.60–1.29)		1.45 (1.21–1.74)	
	No	1.00		1.00		1.00	
Spinal fracture							
Male			<0.001 *		<0.001 *		0.229
	Yes	3.00 (1.71–5.23)		2.37 (1.48–3.80)		1.35 (0.83–2.18)	
	No	1.00		1.00		1.00	
Female			<0.001 *		<0.001 *		<0.001 *
	Yen	1.78 (1.41–2.23)		1.33 (1.16–1.53)		1.27 (1.12–1.44)	
	No	1.00		1.00		1.00	

* Cox-proportional hazard regression model, significance at *p* < 0.05; † stratified model for age, sex, income, region of residence, hypertension, diabetes mellitus, and dyslipidemia; ‡ adjusted model for ischemic heart disease, cerebral stroke, depression, and osteoporosis histories.

## Data Availability

Distribution of the data by the researcher is not legally allowed. All the data are available from the National Health Insurance Sharing Service (NHISS) database (https://nhiss.nhis.or.kr/, accessed on 1 March 2017). The NHISS allows access to all the data to any researcher who promises to follow research ethics standards for a fee. If you want to access the data of this article, you can download them from the website after agreeing to follow research ethics standards.
